# An analysis of the transcriptome of *Teladorsagia circumcincta: *its biological and biotechnological implications

**DOI:** 10.1186/1471-2164-13-S7-S10

**Published:** 2012-12-07

**Authors:** Ranjeeta Menon, Robin B Gasser, Makedonka Mitreva, Shoba Ranganathan

**Affiliations:** 1 Department of Chemistry and Biomolecular Sciences, Macquarie University, Sydney, New South Wales 2109, Australia; 2 Department of Veterinary Science, The University of Melbourne, 250 Princes Highway, Werribee, Victoria 3030, Australia; 3 The Genome Institute, Washington University School of Medicine, 444 Forest Park Boulevard, St. Louis, MO 63108, USA; 4 Department of Genetics, Washington University School of Medicine, 444 Forest Park Boulevard, St. Louis, MO 63108, USA; 5 Department of Biochemistry, Yong Loo Lin School of Medicine, National University of Singapore, 8 Medical Drive, Singapore 117597

## Abstract

**Background:**

*Teladorsagia circumcincta *(order Strongylida) is an economically important parasitic nematode of small ruminants (including sheep and goats) in temperate climatic regions of the world. Improved insights into the molecular biology of this parasite could underpin alternative methods required to control this and related parasites, in order to circumvent major problems associated with anthelmintic resistance. The aims of the present study were to define the transcriptome of the adult stage of *T. circumcincta *and to infer the main pathways linked to molecules known to be expressed in this nematode. Since sheep develop acquired immunity against *T. circumcincta*, there is some potential for the development of a vaccine against this parasite. Hence, we infer excretory/secretory molecules for *T. circumcincta *as possible immunogens and vaccine candidates.

**Results:**

A total of 407,357 ESTs were assembled yielding 39,852 putative gene sequences. Conceptual translation predicted 24,013 proteins, which were then subjected to detailed annotation which included pathway mapping of predicted proteins (including 112 excreted/secreted [ES] and 226 transmembrane peptides), domain analysis and GO annotation was carried out using InterProScan along with BLAST2GO. Further analysis was carried out for secretory signal peptides using SignalP and non-classical sec pathway using SecretomeP tools.

For ES proteins, key pathways, including Fc epsilon RI, T cell receptor, and chemokine signalling as well as leukocyte transendothelial migration were inferred to be linked to immune responses, along with other pathways related to neurodegenerative diseases and infectious diseases, which warrant detailed future studies. KAAS could identify new and updated pathways like phagosome and protein processing in endoplasmic reticulum. Domain analysis for the assembled dataset revealed families of serine, cysteine and proteinase inhibitors which might represent targets for parasite intervention. InterProScan could identify GO terms pertaining to the extracellular region. Some of the important domain families identified included the SCP-like extracellular proteins which belong to the pathogenesis-related proteins (PRPs) superfamily along with C-type lectin, saposin-like proteins. The 'extracellular region' that corresponds to allergen V5/Tpx-1 related, considered important in parasite-host interactions, was also identified.

Six cysteine motif (SXC1) proteins, transthyretin proteins, C-type lectins, activation-associated secreted proteins (ASPs), which could represent potential candidates for developing novel anthelmintics or vaccines were few other important findings. Of these, SXC1, protein kinase domain-containing protein, trypsin family protein, trypsin-like protease family member (TRY-1), putative major allergen and putative lipid binding protein were identified which have not been reported in the published *T. circumcincta *proteomics analysis.

Detailed analysis of 6,058 raw EST sequences from dbEST revealed 315 putatively secreted proteins. Amongst them, C-type single domain activation associated secreted protein ASP3 precursor, activation-associated secreted proteins (ASP-like protein), cathepsin B-like cysteine protease, cathepsin L cysteine protease, cysteine protease, TransThyretin-Related and Venom-Allergen-like proteins were the key findings.

**Conclusions:**

We have annotated a large dataset ESTs of *T. circumcincta *and undertaken detailed comparative bioinformatics analyses. The results provide a comprehensive insight into the molecular biology of this parasite and disease manifestation which provides potential focal point for future research. We identified a number of pathways responsible for immune response. This type of large-scale computational scanning could be coupled with proteomic and metabolomic studies of this parasite leading to novel therapeutic intervention and disease control strategies. We have also successfully affirmed the use of bioinformatics tools, for the study of ESTs, which could now serve as a benchmark for the development of new computational EST analysis pipelines.

## Introduction

Parasitic nematodes have a free-living state with their growth and survival controlled by the surrounding environment, especially by factors such as temperature and moisture.

*Teladorsagia circumcincta *is a key parasite that affect small ruminants in many countries around the world. Its lifecycle is direct and is similar to a number of gastrointestinal strongylid nematodes [1]. In brief, eggs released in faeces develop, and first-stage larvae (L1s) hatch usually within a day. L1s develop through to infective third-stage larvae (L3s) within about a week. L3s on pasture are ingested by the ruminant host, within which they exsheath in the rumenoreticulum and then pass to the abomasum to enter gastric glands and moult to fourth-stage larvae (L4). After this histotrophic phase, these larvae develop to adult female and male worms which reproduce.

*T. circumcincta *can be a major cause of economic loss due to poor productivity of ruminants, such as sheep and goats, failure to thrive and deaths, mainly in lambs [2,3]. Together with other trichostrongylid nematodes, this parasite is usually controlled using a combination of anthelmintic treatment and management strategies. The emergence of resistance in trichostrongylids to the three main classes of anthelmintic drugs, including benzimidazoles (white drenches), imidazothiazoles/tetrahydropyrimidines (yellow/pink drenches) and macrocyclic lactones (clear drenches) compromises effective control. Improved insights into the molecular biology of these parasites have the potential to support the development of alternative methods of parasite control, in order to circumvent these resistance problems. Vaccination is considered by some researchers [4] to be a possible alternative approach to anthelmintic treatment, but attempts to develop a practical, commercial vaccine have been unsuccessful to date, likely because of a lack of detailed understanding of the immuno-molecular biology of the parasites, host-parasite interactions and disease. In spite of the economic significance of *T. circumcincta*, particularly in lambs, our understanding of the spectrum of antigens and immunogens involved in immune responses is still limited [5-7]. Nonetheless, there is evidence that excretory/secretory (ES) molecules are intimately involved in inducing and/or modulating the host's immune response [8], and it has been proposed that some of them are immunogens which could serve as potential vaccine targets [9,10].

Antigenic or immunogenic molecules can be studied using a range of immunochemical or proteomic approaches [11], and transcriptomic studies can strengthen such investigations by providing annotated datasets to allow the identification and classification of such key molecules. For instance, transcriptomic study of *T. circumcincta *has identified a number of components, including N-type and C-type single domain, activation-associated secreted proteins (ASPs) [5]. Preliminary evidence showed that the proteins inferred to represent the secretome in *T. circumcincta *larvae were associated with specific antibody responses in sheep against this parasite. These proteins might be incorporated into a vaccine for immunizing sheep to combat the Teladorsagiosis disease [12]. Importantly, N-type and C-type single domain activation-associated secreted proteins (ASPs) and *T*. *circumcincta *apyrase-1 (Tci-APY-1) in excretory/secretory products of L4s of *T. circumcincta*, identified also in transcriptomic studies [5,13], have been demonstrated to be targets for early, specific IgA responses in infected sheep [5]. In addition, it has been reported that Tci-MIF-1, a macrophage migration inhibitory factor (MIF)-like molecule with tautomerase activity, might influence both host immune responses and nematode physiology [14]. Therefore, a detailed exploration of the transcriptome of *T. circumcincta *will provide a vital insight into the molecular biology of this parasite and should also provide a basis for studying parasite-host interactions and disease as well as parasite development and reproduction, with a view towards establishing new methods of prevention, treatment or control. Extending previous studies of strongylid nematodes [15-18], we report the first comprehensive analysis of the transcriptome from the adult stage of *T. circumcincta*, with an emphasis on characterization of molecules inferred to be ES proteins.

## Materials and methods

The ESTs (NCBI EST database accession numbers SRR328404 and SRR328405) was generated by LS454 RNAseq sequencing of *T. circumcincta *2284716780 fragment cDNA library using 454 GS FLX Titanium instrument. The dataset was initially assembled and annotated using different tools. Initially, all ESTs were pre-processed (using SeqClean [19] and RepeatMasker (Smit AFA & Green P)), for the removal of low-quality regions and consensus sequence generation using the Contig Assembly Program CAP3 which was followed by assembly [20]. This step was followed by ESTScan [21] translation of the contiguous sequences (contigs) into peptides, which were then characterized *via *InterProScan [22] domain/motifs. Gene ontologies were inferred using BLAST2GO (V 2.3.5) [23], from Gene Ontology (MySQL-DB-data release go_200903) and InterProScan. Peptides predicted were also compared, using BLASTP, with data in the non-redundant protein sequence database from National Centre for Biotechnology Information (NCBI). The peptides were mapped to respective pathways in *C. elegans *using KOBAS [24] (KEGG [25] Orthology-Based Annotation System, KOBAS-1.1.0). The results were compared with pathway mapping using KAAS [26]. Similarity searches were done for protein databases for 'parasitic nematodes' and 'non-nematodes' generated in-house. Homologues/orthologues were identified *via *comparisons against WormBase using BLASTX. In addition, data for *C. elegans*, including RNA interference (RNAi), gene ontology, pathway and domain analyses were used for functional annotation.

The program SimiTri [27] was used for the comparison of inferred amino acid sequence data for *T. circumcincta *with those available for *C. elegans*, parasitic nematode and other organisms in public databases. SimiTri provides a two-dimensional display of relative similarity relationships among three different datasets. ES proteins were predicted using SignalP [28] to infer the presence of secretory signal peptides and signal anchors in predicted proteins. SecretomeP [29] was also used to predict proteins involved in a non-classical secretory pathway. Transmembrane proteins were predicted using TMHMM [30], a hidden Markov model-based program. Predicted proteins lacking transmembrane domains were subjected to further annotation using data available in Wormpep [31].

## Results

### cDNA analysis

From a total of 407,357 raw ESTs representing *T. circumcincta*, we obtained 366,897 high quality ESTs (Table 1), which ranged from 100-415 bp in length (mean: 206 bp; standard deviation: 43 bp). After clustering and assembly, the mean length of contigs increased to 360 bp (standard deviation: 173 bp). The G+C content of the coding sequence was 42%, consistent with other strongylid nematodes [15,32]. The assembly of the 366,897 ESTs yielded 39,852 representative sequences (22,382 contigs and 17,470 singletons; Table 1), of which 24,013 (60.3%) had open reading frames (ORFs). Similarity searches of these representative sequences identified 19,540 (49%) homologues in *C. elegans*, 32,476 (81.5%) in other parasitic nematodes and 13,064 (32.78%) in organisms other than nematodes.

**Table 1:** Preliminary analysis of the 407357 *T. circumcincta *ESTs.

*T. circumcincta ESTs*	Numbers (percentage)
Raw sequences obtained	407357
Cleaned sequences	366897 (90.06)
Clusters of multiple sequences (contigs)	22382 (5.4)
Clusters of singletons	17470 (4.2)
Total rESTs	39852 (9.7)
Putative peptides	24013 (60.25 % rESTs)

**E/S proteins (cut-off: 0.5) **	112

The contigs and singletons generated by preprocessing, overall representative ESTs (rESTs), peptides from conceptual translation and putative excretory-secretory (E/S) proteins identified are shown.

Of the 6,628 (16.63 %) well-characterized molecules known to be associated with various biological processes (Additional File 1). Similarly, a comparative analysis of all 39,852 rESTs was also carried out using data from various nematodes (such as *Haemonchus contortus*, *Necator americanus*, *Nippostrongylus brasiliensis*, *Ostertagia ostertagi*, *Oesophagostomum dentatum*, *Ancylostoma caninum*, *Dictyocaulus viviparus*) [32]; Mitreva et al., 2006) to explore gene conservation within clade V (Additional File 2). The analysis showed that 13,531 ESTs (33.95%) had significant sequence similarity to molecules from the members of clade V at an e-values cut-off of 1e-05.

6156 of them were mapped to 234 KEGG pathways of the homologues identified in *C. elegans*. *Oxidative phosphorylation *(n = 357) and *Peptidases *(n = 277 peptidases) were the highest represented according to the number of peptides mapped. Other groups of molecules were mapped to metabolic pathways such as *glycine, serine and threonine metabolism *(n = 93), *insulin signaling pathway *(n = 68), *signal transduction mechanisms *(n = 54), *N-glycan biosynthesis *(n = 33), *galactose metabolism *(n = 31), *GnRH signaling pathway *(n = 13), *aminosugars metabolism *(n = 11), *linoleic acid metabolism *(n = 5), *immune and complement and coagulation cascades *(n = 4). A list of the KEGG pathways and the corresponding rESTs is provided as supplementary information (Additional File 3).

### Peptides/Proteins

Of the 39,852 rESTs, 24,013 were inferred to have open reading frame (ORFs). 6,470 sequences mapped to 309 KEGG pathways, with the top 30 'highly represented' pathways categorized by the number of peptides mapped, presented in Table 2. The main KEGG pathways represented were the *peptidases *(n = 254) and *ribosomal protein assembly pathway *(n = 220). Other highly represented pathways by the peptides include *oxidative phosphorylation *(n = 187) and *chaperones and folding catalysts *(n = 144). Peptides were mapped to several pathways, including *purine metabolism *and *glycolysis/gluconeogenesis*. We have also compared our results by mapping the sequences using KAAS where 2,897 sequences were characterized as belonging to 257 pathways, with 30 'highly represented' pathways, categorized according by the number of peptides mapped, are presented in Table 3. The main KAAS pathways represented were *Huntington's disease *(n = 91) and *oxidative phosphorylation *(n = 84). Other highly represented pathways include the *ribosomal protein assembly pathway *(n = 80), *ubiquitin mediated proteolysis *(n = 33) and *glycolysis/gluconeogenesis *(n = 29).

**Table 2:** Top 30 metabolic pathways mapped by Kyoto Encyclopedia of Genes and Genomes in *T. circumcincta *protein sequences

KEGG PATHWAY	SEQUENCE COUNT
Peptidases	254
Ribosome	220
Oxidative phosphorylation	187
Other enzymes	168
Chaperones and folding catalysts	144
Cytoskeleton proteins	109
Protein kinases	108
Purine metabolism	102
Translation factors	96
Ubiquitin enzymes	90
Proteasome	89
Starch and sucrose metabolism	86
Pyruvate metabolism	86
Glycolysis/Gluconeogenesis	83
Fatty acid metabolism	83
Lysine degradation	78
Valine, leucine and isoleucine degradation	76
Tryptophan metabolism	72
Aminoacyl-tRNA biosynthesis	69
Insulin signaling pathway	68
GTP-binding proteins	68
Citrate cycle (TCA cycle)	68
Regulation of actin cytoskeleton	65
Propanoate metabolism	64
Cell cycle	64
Carbon fixation	64
Focal adhesion	62
Ubiquitin mediated proteolysis	60
Fructose and mannose metabolism	60
Butanoate metabolism	59

**Table 3:** Top 30 metabolic pathways mapped by KAAS in *T. circumcincta *protein sequences

KEGG PATHWAY	PROTEINS
Huntington's disease	91
Oxidative phosphorylation	84
Ribosome	80
Spliceosome	79
Alzheimer's disease	72
Parkinson's disease	70
Purine metabolism	56
Pyrimidine metabolism	51
Cell cycle	34
Ubiquitin mediated proteolysis	33
Proteasome	33
Lysosome	33
Endocytosis	32
Cell cycle - yeast	31
Peroxisome	30
Glycolysis/Gluconeogenesis	29
Pathways in cancer	28
Aminoacyl-tRNA biosynthesis	28
DNA replication	26
Valine, leucine and isoleucine degradation	25
Regulation of actin cytoskeleton	25
Citrate cycle (TCA cycle)	25
Vibrio cholerae infection	23
Fatty acid metabolism	23
Amino sugar and nucleotide sugar metabolism	23
RNA degradation	22
Nucleotide excision repair	21
Lysine degradation	21
RNA polymerase	20
Meiosis - yeast	20

Peptides were also mapped to several other pathways, including *purine metabolism *and *pyrimidine metabolism*, pathways in *cancer, cysteine and methionine metabolism*, *glycolipid metabolism *and *glutathione metabolism*. Among the highly represented pathways, both KEGG and KAAS identified *oxidative phosphorylation*, *purine metabolism*, *glycolysis/gluconeogenesis *and *ribosomal protein assembly *pathways. We could identify GO terms using InterProScan for 24,013 proteins with 3,801 being assigned as involved in biological process (BP), 5,220 as associated with molecular function (MF) and 1,862 as part of the cellular component (CC) (Additional File 4). The analysis revealed that *oxidation reduction *(GO:0055114) and *metabolic process *(GO:0008152) were the most common GO categories representing biological processes. The highest represented GO terms in molecular function were *binding *(GO: 0005488) *and oxidoreductase activity *(GO:0016491). Whereas in cellular component, the highly represented GO terms were *ribosome *(GO:0005840) and *membrane *(GO:0016020). With 138 protein entries, the *protein kinase-like domain *family of proteins was the most represented, followed by *SCP-like extracellular domain *family, with 126 protein entries. Other highly represented group of domains are the *NAD(P)-binding domain*, *allergen V5/Tpx-1 related domain *and *transthyretin-like domain *(Table 4).

**Table 4:** Top 30 domain description for the protein sequences

Description	InterProscan ID	Protein sequences
Protein kinase-like domain	IPR011009	138
SCP-like extracellular	IPR014044	126
NAD(P)-binding domain	IPR016040	96
Allergen V5/Tpx-1 related	IPR001283	95
Transthyretin-like	IPR001534	88
C-type lectin fold	IPR016187	85
C-type lectin	IPR001304	78
C-type lectin-like	IPR016186	71
Nucleotide-binding, alpha-beta plait	IPR012677	71
Serine/threonine-protein kinase-like domain	IPR017442	69
Metridin-like ShK toxin	IPR003582	67
RNA recognition motif, RNP-1	IPR000504	64
Peptidase C1A, papain	IPR013128	59
Thioredoxin-like fold	IPR012336	57
WD40 repeat, subgroup	IPR019781	56
WD40 repeat-like-containing domain	IPR011046	56
WD40/YVTN repeat-like-containing domain	IPR015943	54
Thioredoxin fold	IPR012335	53
Pyridoxal phosphate-dependent transferase, major domain	IPR015424	52
Heat shock protein Hsp20	IPR002068	51
Protein-tyrosine phosphatase, receptor/non-receptor type	IPR000242	50
EF-hand-like domain	IPR011992	49
Peptidase A1	IPR001461	48
Tyrosine-protein kinase	IPR020685	47
Peptidase C1A, papain C-terminal	IPR000668	47
Peptidase aspartic	IPR021109	45
Short-chain dehydrogenase/reductase SDR	IPR002198	45

### Secretome

We inferred 112 excreted/secreted proteins from the present data set of 39,852 rESTs (Additional File 5). Six Transthyretin proteins followed by three saposin-like protein1 from *A. caninum*, three SXC1 (Six Cysteine Motif) proteins of *O. ostertagi*, two C-type single domain activation associated secreted protein ASP3 precursor from *O. ostertagi *were identified. Two C-type lectin-1 proteins represented in *Heligmosomoides polygyrus *and FMRFamide-like prepropeptide from *Oesophagostomum dentatum *one each of globin-like protein and putative L3 ES proteins of *O. ostertagi*, the bovine parasite which is closely related to *T. circumcincta *[33] were also identified. Neuropeptides or neuropeptide precursor molecules were represented among the annotated ES dataset.

Upon detailed annotations of the 112 adult secreted proteins, few novel proteins such as SXC1, protein kinase domain containing protein, trypsin family protein, TRYpsin-like protease family member (try-1), putative lipid binding protein were also identified. These novel proteins were not reported in the *T. circumcincta *proteomics analysis [12,34] (Additional File 6). Subsequent detailed annotation of 226 transmembrane proteins helped in the identification of SXC1 (Six Cysteine Motif) proteins of *O. ostertagi*, putative L3 ES protein (*O. ostertagi*), putative major allergen (*Brugia malayi*). The details of these proteins are listed in Additional File 7.

**Figure 1:**
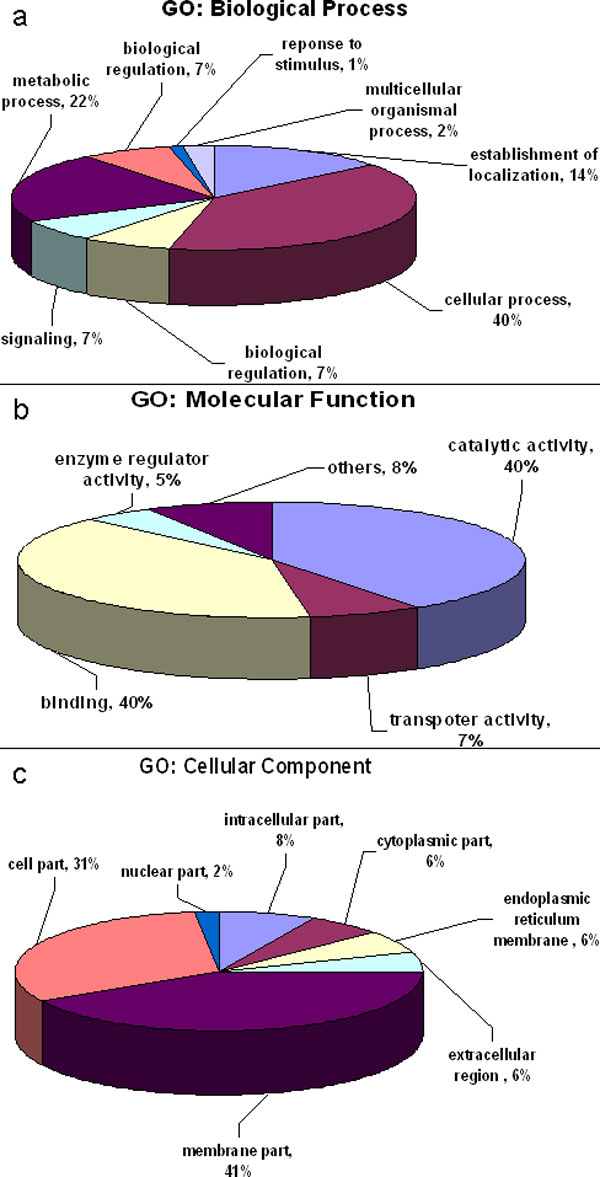
**Putative excretory-secretory proteins and Gene Ontology (GO) terms identified**. Percentages show the annotated categories a. Cellular Component, b. Molecular Function and c. Biological Process.

We were able to functionally assign GO terms to 112 putative ES proteins with 50 being assigned as involved in biological process (BP), 81 as associated with molecular function (MF). The GO annotation summary with biological process, cellular component and molecular function details is provided in Figure 1. *Oxidation reduction *(GO:0055114) and *transmembrane transport *(GO:0055085) were the most common GO categories representing biological processes. The highest represented GO terms in molecular function were *binding *(GO: 0005488) and *catalytic activity *(GO: 0003824), known for their role in the identification of vaccine candidates or drug discovery. Additional File 8 gives a list of GO mappings consigned to ES protein data is provided in. 63 KEGG pathways showed mapping to 90 sequences with the top 30 'highly represented' pathways, categorized according to the number of putative ES proteins mapped, are presented in Table 5. *Protein kinases *(n = 3) and *oxidative phosphorylation *(n = 3) were the main KEGG pathways that mapped to the ES protein sequences.

**Table 5:** Top 30 selected metabolic pathways in excretory-secretory proteins mapped using KEGG database

KEGG PATHWAY	ES Proteins
Protein kinases	3
Oxidative phosphorylation	3
Long-term depression	3
Glycerophospholipid metabolism	3
Arachidonic acid metabolism	3
VEGF signaling pathway	2
Purine metabolism	2
Protein folding and associated processing	2
Peptidases	2
MAPK signaling pathway	2
Linoleic acid metabolism	2
GnRH signaling pathway	2
Glycolysis/Gluconeogenesis	2
Glutathione metabolism	2
Fc epsilon RI signaling pathway	2
Ether lipid metabolism	2
Cytoskeleton proteins	2
CAM ligands	2
alpha-Linolenic acid metabolism	2
Wnt signalling pathway	1
Urea cycle and metabolism of amino groups	1
Ubiquitin mediated proteolysis	1
Ubiquitin enzymes	1
Tyrosine metabolism	1
Type II diabetes mellitus	1
Translation factors	1
Transcription factors	1
Tight junction	1
TGF-beta signaling pathway	1
Signal transduction mechanisms	1
Other enzymes	4

Few other highly represented pathways by the ES proteins include the *glycerophospholipid metabolism *(n = 3), *long-term depression *(n = 3), *glycolysis/gluconeogenesis *(n = 2). Several pathways including *purine metabolism*, *protein folding and associated processing*, *MAPK signaling pathway*, *linoleic acid metabolism*, *GnRH signaling pathway *and *glutathione metabolism *were mapped by ES protein sequences. The list of KEGG pathways for ES proteins is available from Additional File 9.

55 KEGG pathways contained 85 sequences using KAAS with the top 30 'highly represented' pathways, categorized by the number of peptides mapped, are presented in Table 6. *Glycerophospholipid metabolism *(n = 3) and *oxidative phosphorylation *(n = 3) were the main KEGG pathways that mapped to the sequences. Few other highly represented pathways by ES proteins included *long-term depression *(n = 3) and *Wnt signaling pathway *(n = 2). ES proteins were mapped to several pathways such as *MAPK signaling pathway*, *linoleic acid metabolism*, *GnRH signaling pathway*, *glutathione metabolism *and *TGF-β signaling pathway*. The KEGG pathways with the corresponding ES proteins are provided in Additional File 10.

**Table 6:** Pathway Analysis of secreted proteins using KAAS

KEGG PATHWAY	ES Proteins
Glycerophospholipid metabolism	3
Oxidative phosphorylation	3
Vascular smooth muscle contraction	3
Long-term depression	3
Arachidonic acid metabolism	3
Alzheimer's disease	3
Wnt signaling pathway	2
VEGF signaling pathway	2
Tight junction	2
TGF-beta signaling pathway	2
Parkinson's disease	2
Oocyte meiosis	2
Meiosis - yeast	2
MAPK signaling pathway	2
Lysosome	2
Linoleic acid metabolism	2
Huntington's disease	2
GnRH signaling pathway	2
Glutathione metabolism	2
Fc epsilon RI signaling pathway	2
Ether lipid metabolism	2
Cell cycle - yeast	2
Axon guidance	2
alpha-Linolenic acid metabolism	2
Pyruvate metabolism	1
Glycolysis/Gluconeogenesis	1
Carbon fixation in photosynthetic organisms	1
Citrate cycle	1
Vibrio cholerae infection	1
Ubiquitin mediated proteolysis	1

Table 7 gives the top 20 representative protein families with *metridin-like ShK toxin *as the highly represented family of proteins, comprising of 14 ES protein entries. Followed by *transthyretin-like *family of proteins, comprising 11 ES protein entries. *C-type lectin*, *saposin-like domain *and *SCP-like extracellular domain superfamily of the pathogenesis-related proteins *(PRPs) [35,36] were the few other well-represented domain families in the present datasets. SecretomeP identified 615 sequences as non-classical secreted proteins at a cut-off value of 0.9. The detailed annotation of 615 secreted proteins revealed 62 KEGG pathways mapped by 105 sequences (Additional File 11) with the top highly represented pathways presented in Table 8.

**Table 7:** Top 20 protein families of known function found in excretory-secretory proteins

Description	ES sequences	Type	Interproscan ID
Metridin-like ShK toxin	14	Domain	IPR003582
Transthyretin-like	11	Family	IPR001534
SCP-like extracellular	7	Domain	IPR014044
Saposin-like	7	Domain	IPR011001
C-type lectin	7	Domain	IPR001304
C-type lectin fold	6	Domain	IPR016187
C-type lectin-like	6	Domain	IPR016186
Proteinase inhibitor I2, Kunitz metazoa	5	Domain	IPR002223
Protein kinase-like domain	4	Domain	IPR011009
Major facilitator superfamily, general substrate transporter	4	Domain	IPR016196
Destabilase	3	Family	IPR008597
Allergen V5/Tpx-1 related	3	Family	IPR001283
Tyrosine-protein kinase	3	Region	IPR020685
Phospholipase A2	2	Family	IPR016090
Thioredoxin-like fold	2	Domain	IPR012336
Thioredoxin fold	2	Domain	IPR012335
Globin	2	Domain	IPR012292
Serine/cysteine peptidase, trypsin-like	2	Domain	IPR009003
Saposin B	2	Domain	IPR008139
Protein of unknown function DUF148	2	Domain	IPR003677

**Table 8:** Top 30 Pathway analysis of secreted proteins obtained from SecretomeP

KEGG PATHWAY	ES Proteins
Translation factors	6
Oxidative phosphorylation	4
Cell cycle	4
Regulation of actin cytoskeleton	3
Protein kinases	3
Progesterone-mediated oocyte maturation	3
Peptidases	3
DNA polymerase	3
Chaperones and folding catalysts	3
Ubiquitin mediated proteolysis	2
Ubiquitin enzymes	2
Transcription factors	2
Tight junction	2
RNA polymerase	2
Ribosome	2
Reductive carboxylate cycle (CO2 fixation)	2
Pyruvate metabolism	2
Pores ion channels	2
mTOR signaling pathway	2
MAPK signaling pathway	2
Glutathione metabolism	2
General function prediction only	2
Gap junction	2
Fatty acid metabolism	2
Fatty acid biosynthesis	2
Cytoskeleton proteins	2
Citrate cycle (TCA cycle)	2
Cell cycle - yeast	2
Arginine and proline metabolism	2
Other enzymes	2

*Translation factors *and *oxidative phosphorylation *were the main KEGG pathways that mapped to the sequences. *Protein kinases, peptidases, chaperones *and *folding catalysts *are among other well represented pathways by ES proteins. The analysis of 6,058 raw EST sequences from dbEST with an overlap of 20.3% with the cDNA resulted in 745 contigs and 1,696 singletons, where 2,242 had ORFs.

We could identify 315 putatively secreted proteins and 183 transmembrane proteins. An in-depth analysis of secreted proteins, identified 11 *C-type single domain activation associated secreted protein *(ASP3) precursors (*O. ostertagi*), ten *ancyclostoma-secreted protein-like proteins *(*O. ostertagi*), five *cathepsin B-like cysteine proteases *(*O. ostertagi*), one *cathepsin L cysteine protease *(*H. contortus*), three *cysteine proteases*, four *precursor transthyretin like protein 1 *(*O. ostertagi*), six *putative L3 ES proteins *(*O. ostertagi*), five *saposin-like protein 1 *(*A. caninum*), three *secreted cathepsin F *(*T. circumcincta*), two *SXC1 proteins *(*O. ostertagi*), three *TransThyretin-related proteins*, two *venom-allergen-like proteins*.

## Discussion

**Figure 2:**
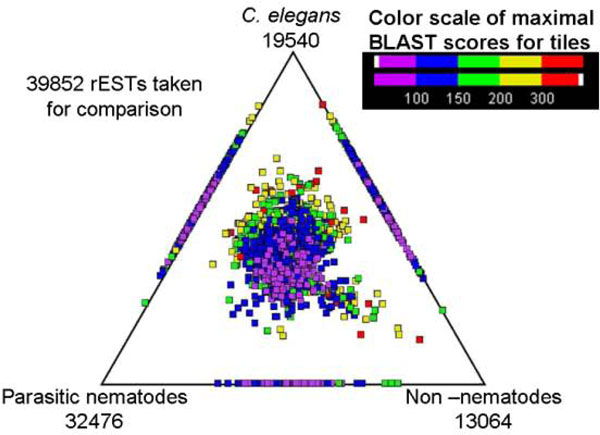
**Comparison of *T. circumcincta *rESTs with *C. elegans*, other parasitic nematodes and organisms other than nematodes, from SimiTri analysis**. The numbers at each vertex indicate rESTs matching that specific database.

In the absence of a genomic sequence for *T. circumcincta*, 407,357 raw EST sequences were analysed to obtain quality ESTs with a sequencing success of 90.06% which is consistent with previous studies [15,34,37]. To infer the proteome for *T. circumcincta*, all rESTs were then subjected to analyses against three databases containing protein sequences. Data were compared with protein sequences available for (i) *C. elegans *(from WORMPEP v.182 Wombase([http://wormbase.org/])), (ii) parasitic nematodes (available protein sequences and peptides from conceptually translated ESTs) and (iii) organisms other than nematodes (from NCBI non-redundant protein database) [38]. Three-way comparison of *T. circumcincta *rESTs with homologues from *C. elegans*, WORMPEP and parasitic nematodes have been figuratively presented (Figure 2) using SimiTri.

Some of the proteins predicted to be parasite- or nematode-specific were identified by similarity searches of rESTs and these proteins in parasitic nematodes were either absent from or very different from the corresponding molecules in their host(s).

Comparative analysis was carried out to identify homologues in *C. elegans*, the best characterized nematode in relation to its genome, genetics, biology, physiology, biochemistry as well as the localization and functions of molecules Wormbase [39]. This study showed that 7,537 of them were mapped to key biological pathways including *oxidative phosphorylation*, *peptidases *and the *ribosomal protein assembly pathway*. *Oxidative phosphorylation *relates to genes that encode NADH dehydrogenases, succinate dehydrogenases, cytochrome c oxidases, cytochrome c reductases, ATPases and ATP synthases (complexes I-V) [40]. Several *peptidases *are known to play a vital role in the moulting process [41], these include metallo-peptidases that might be candidates for chemotherapeutic interventions [42-45]. The *ribosomal protein assembly pathway *is composed of genes that encode various proteins of the ribosomal subunits. These proteins are closely related functionally and need to interact with each other physically to form a large protein complex known as the ribosome [40]. Other pathways represented include the *carbon fixation pathways*. Several enzymes in nematodes map to KEGG carbon fixation pathways [http://www.genome.jp/kegg-bin/show_pathway?category=Nematodes&mapno = 00720], which refer to normal energy pathways such as *glycolysis, gluconeogenesis *(which is actually carbon fixing) and *tricarboxylic acid cycle*.

The pathways identified using KOBAS such as *TGF-β signaling pathway *and *insulin signaling pathway *trigger an ''alternative'' developmental pathway and regulate the transition of environmental stress on *C. elegans *in the first larval stage of its life cycle [46,47]. The disruption of both insulin-like and DAF-7 transforming growth factor (TGF)-β signalling pathways causes developmental arrest [48,49]. Abundant levels of transcription of GTP-CH transcripts in some parasitic species could be associated with production of serotonin to regulate these processes, in a way that is similar to that of *C. elegans*, if a TGF-β pathway does indeed regulate developmental events in parasitic nematodes [34]. These areas are of great interest and deserve detailed investigation, particularly given that molecules representing the TGF-β pathway have been described for a number of parasitic nematodes such as *B. pahangi*, *B. malayi *and *P. trichosuri *[50-52].

Proteins expected to play critical roles in host-parasite interactions including immune responses are predicted to be involved in antigen processing and presentation or complement and coagulation cascades.

Nematode enzymes mapped to known human disease pathways such as *Huntington's disease, Alzheimers disease, Parkinson's disease *and *Vibrio cholerae infection*. The neurological disorder pathways are known to describe the morbidity and depression associated with helminthic infections. The *Vibrio cholera *infection pathway supports this parasite being similar to gastrointestinal strongylid nematodes.

Clearly, much more work is required to establish the functional roles of such proteins in the parasite and/or the host and also to identify essential proteins required in each pathway, even though they are not well represented. Some of the proteins are inferred to be excreted/secreted from the nematode. These include *serine proteinase inhibitors *and *cathepsin B-like cysteine proteases *which are proposed to interfere with the immune system at the antigen processing and presentation stages, thereby, to interrupt the cytokine network and to down-regulate inflammation [53]. Families of proteins considered as important targets for parasite invention and control were also identified represented by *serine, cysteine *as well as *proteinase inhibitors *which are also supported by domain analysis [54-56]. The proteinase inhibitors might protect the parasite against digestion by endogenous or host-derived proteinases [53].

Of the 39,852 rESTs, 24,013 were inferred to have open reading frame (ORFs). The most represented domain family of proteins were the *protein kinase-like *and the *SCP-like extracellular domains*, followed by *NAD(P)-binding domain, allergen V5/Tpx-1 related domain *and *transthyretin-like domain*. Analysis of several protein and protein domains present in *C. elegans *[57] revealed that protein kinases comprise the second largest family of protein domains in worms. Protein kinases are required for the existence of multicellular organisms and are likely to be involved in the complex signal transduction pathways including cell-substratum and cell-cell adhesion, transmembrane signaling in response to humoral factors and cell survival or programmed cell death. Other protein kinases provide signals that regulate metazoan-specific transcription factors, particularly those containing Zn-finger domains [58].

*SCP/TAPS family *members belong to the *cysteine-rich secretory protein *(CRISP) and have been identified in various eukaryotes. They also seem to have some biological roles linked with the member proteins within this superfamily [59].

The *sperm-coating protein (SCP)-like *extracellular proteins, also called SCP/Tpx-1/Ag5/PR-1/Sc7, play major biological roles in the host-pathogen interplay [60] along with other groups of proteins [61] . NADP^+ ^plays a vital role in developmental process and also acts as a reducing agent in anabolism along with NAD^+^, a coenzyme involved in key pathways like *glucose metabolism *and *fatty acid synthesis *[62]. In *Strongyloidae*, the *allergen V5/Tpx-1 related domain *is considered as one of the most abundant InterPro domain that may be important in parasitism [32]. It symbolizes various members such as the *ancylostoma-secreted or activation-associated proteins *(ASPs) that belong to the pathogenesis-related protein (PRP) superfamily [35]. The *transthyretin-like domain*, an abundant nematode-specific motif [63] was recently identified as being abundantly transcribed in the transcriptome of *B. malayi *[64]. *Lectins *are carbohydrate binding proteins and the CLec fold constitutes a general ligand (including protein)-binding motif [65].

The vertebrate immune cell signalling and trafficking, activation of innate immunity in both vertebrates and invertebrates and venom-induced haemostasis, have the involvement of C-type lectins [66]. *Metridin-like ShK toxin domains *are highly represented in the Strongylida [32]. Though the specific function of these proteins are not known, they are assumed to be involved in defense or digestion [67]. *WD40 repeats *(also known as WD or beta-transducin repeats) are involved in signal transduction and transcription regulation along with cell-cycle control and apoptosis [68,69].

*Heat shock proteins*, such as HSP-20 are reported to be present in the parasitic nematode, *H. contortus *(barber's pole worm) which afflicts small ruminant species and in the adult stage of *A. caninum *and other nematodes including the bovine lungworm *Dictyocaulus viviparus *and the common roundworm of canids *Toxocara canis*. The expression of this molecule was shown not to be controlled by heat shock treatment [70].

*'EF-hand' domains *are involved in protein-protein interactions regulated by various specialized systems (e.g., Golgi system, voltage dependent calcium channels and calcium transporters) [71]. The maturation of the nervous system and the formation of ciliated sensory neurons require both EF-hand and WD40 proteins in *C. elegans *[72,73]. *Major sperm proteins (MSPs)*, a large protein family, are known to be largely involved in nematode sperm motility [74,75]. MSPs (expressed in recombinant form) have been proposed as vaccine candidates [76]. The entire list of domains and their details are given in Additional File 12. The protein sequences were assigned functionality based on BLASTP against the NR database (Additional File 13). Different classes of proteases are assigned based on the catalytic mechanisms and are named based on their active catalytic centre residues (*aspartic, serine and cysteine proteases*) or after their dependence on co-factors for activity (*metalloproteases*). Of the four classes of proteases aspartic proteases are considered to be the most conserved group.

*Cysteine proteases *are most likely involved in tissue penetration and feeding [77]. *Cysteine, aspartic and metallo-proteases *represented in *N. americanus*, are known to function in a multi-enzyme cascade to digest haemoglobin and other serum proteins [78,79]. *SCP (sperm coating protein)-1 superfamily *members include insect venom allergens, plant pathogenesis family-1 (PR-1) proteins and VAL proteins beside mammalian cysteine-rich sperm proteins (CRISPs). No rational function for this protein family has been demonstrated despite the sequence similarity [8]. *Astacin-like metalloproteases *are vital for establishment of the parasite in the host. *MTP-1 *and *the astacin-like MTP *secreted by infective larvae of hookworms, are primarily reported in *A. caninum *[80-82]. The enzyme *guanosine-50-triphosphate (GTP)-cyclohydrolase *may be involved in larval development [35]. In parasitic nematodes, *astacin-like *molecules are considered to be involved with moulting, tissue penetration and immunomodulation besides feeding [34,80]. They are also anticipated to be vaccine candidates against parasitic nematodes [82,83].

Pathway analysis using KOBAS [24] mapped a total of 6,470 sequences to 309 KEGG pathways. The results were compared by mapping the sequences using KAAS [26], where a total of 2,897 sequences were mapped to 257 KEGG pathways. The perceptive of such mapping in biological pathways will help in identifying vital proteins required in each pathway.

Functionally varied classes of molecules such as digestive enzymes, extracellular proteinases, chemokines, morphogens, cytokines, toxins, hormones, antibodies, antimicrobial peptides included in secretome constitute the entire set of secreted proteins, representing up to 30% of the proteome of an organism [84]. *SXC1 (Six Cysteine Motif) *proteins of *O. ostertagi*, *transthyretin *proteins, *saposin-like protein 1*, *C-type lectin-1*, *globin-like protein*, *Na-ASP-2*, a PR-1 protein from *N. americanus*, *ASP-3 *from *O. ostertagi*, *neuropeptides *and *cytochrome P450s *were also identified from the 112 excreted/secreted proteins inferred from the data set of 39,852 rESTs.

The *SXC domain*, also termed nematode-six cysteine, NC6 [85], was identified in surface coat proteins of the parasitic ascarid *T. canis *[86,87] along with zinc metalloproteases and tyrosinases of *C. elegans*. SXC domains have also been identified in other helminths such as *Ascaris, Brugia*, *Trichuris muris *and *Necator *[88]. The function of the motif is not known but it is suggested that it is involved in protein-protein interactions, particularly those associated with nematode surfaces [89] or that it acts as a signalling ligand [90]. In general, SXC motif containing proteins have a putative secretory signal peptide and are therefore extracellular. The *transthyretin-like *(TTL) gene family, also known as ''family 2'' [91], has been classified as nematode-specific based on the genome-wide study of *C. elegans*. These are the largest conserved nematode-specific gene families, coding for a group of proteins with significant sequence similarity to transthyretins (TTR) and transthyretin-related proteins (TRP) [92]. Transthyretin-like protein families are potential vaccine candidates against human filariasis [93].

As part of transcriptomic analysis of some members of the phylum Nematoda more than 4,000 nematode-specific protein families encoded by nematode-restricted genes were defined with TTL family representing one of the largest [32]. TTL protein domain was represented 185 times in all nematodes studied. This included 18 ttl genes in *O. ostertagi *as a result of protein domain search using the NEMBASE database [92]. The TTL family shows characteristics comparable with those of neuropeptides, i.e., a large protein family with secretion signals and different expression patterns between the members of the family and are likely to play a role in the nervous system of the nematodes [94]. SAPLIPs (saposin-like proteins) are a diverse family of lipid interacting proteins [95] that have six conserved cysteine residues forming three disulfide bridges [95-98]. The majority of Ac-slp-1 is expressed in the L3 and adult worm, although it is detected in RNA from all developmental stages of *A. caninum*.

While the Ac-slp-1 and slp-2 mRNAs are expressed in the intestines of multiple developmental stages of *A. caninum*, suggesting multiple functions in parasite biology, both Ac-SLP-1 and SLP-2 are localized to the intestines and could play a role in parasite feeding. The SLP-1 protein could also interact with host cells [99]. Worm carbohydrates may be masked from host immune cells by parasite C-TLs. Nematode C-TLs may also have roles unconnected with immune evasion [8]. Antigen uptake and presentation, cell adhesion, apoptosis and T cell polarization are the few immune processes in which C-type lectins and galectins are involved [66]. CTLs are perhaps the most prominent in the mammalian immune system. *Heligmosomoides polygyrus*, the natural parasites of mice, are the most widely-studied amongst the parasitic nematodes. Immunological interactions with the host are presumed to be mediated by the new C-type lectins from these rodent parasites which are preferentially expressed by the mature adult stages [100].

Craig *et al*. [101] were able to identify a homologue of a globin-like ES protein from *O. ostertagi *in L4 and adult *T. circumcincta *protein. Adult ES proteins in *O. ostertagi *identified a homologue of an ASP and a vitellogenin [92], which were not identified in *T. circumcincta *ES proteins [101]. However, we have successfully identified a *globin-like protein *and *Na-ASP-2 *- a PR-1 protein from *Necator americanus*) [102] and *ASP-3 *from *O. ostertagi *[103]. ASPs are the members of a group of nematode-specific molecules [5]. Proteins in this family have been identified in a wide range of organisms [35], including human hookworm [104], filarial nematodes [105,106], trichostrongylids such as *H. contortus *[107,108], *schistosomes *[59,109,110] as well as free-living *C. elegans *[111]. It has been suggested that ASPs are key to the transition of nematodes from free-living to the parasitic state [112]. It has also been suggested that they exhibit homology to a diverse, yet evolutionarily-related, group of secreted proteins classified as the SCP/Tpx-1/Ag5/PR-1/Sc7 family [5].

Na-ASP-2 has recently been shown to induce neutrophil chemotaxis *in vitro *and *in vivo *[113], but it remains uncertain if this is a widespread property of VAL homologues [8]. The role of nematode ASPs as valid vaccine candidates has also been investigated [114]. ASPs have been suggested to have the role of allergens [34]. They also have a role in modulation of the host immune response [115], in maintenance of the parasites at their host niche [116,117]and in maintenance and/or exit from arrested development [118]. ASPs are highly represented in EST datasets derived from parasitic stages of *T. circumcincta *and are abundant in the L4 ES proteins of this nematode [34]. Neuropeptide-like proteins have shown to be present in *O. ostertagi *[119]. These intercellular signaling molecules and particularly the FMRFamide-related peptides (FaRPs), have been most widely studied in *Ascaris suum *where they are present throughout the nervous system [34]. Cysteine-rich proteins were highly represented in *T. circumcincta *L4-specific dataset and were suggested to have a role in establishment and immune evasion [113].

Members of the astacin family have a wide range of functions [120] including immunomodulation [121], growth-factor processing, pattern formation in embryos [122], digestion, tissue penetration [80,123] and hatching [124]. Nematode AST-like metalloproteinases play role in stimulating innate and adaptive immune responses early in infection [83]. Cytochrome P450s, the candidate drug-resistance genes, were also identified. These could affect the expression of the functional group 'xenobiotic degradation and metabolism' [6]. We have attempted to integrate the transcriptomics data with the proteomics analysis from previous reports to understand the role of ES proteins in host-parasite interaction (Additional File 6). Kyoto Encyclopedia of Genes and Genomes database (KEGG) was searched with KOBAS and KAAS to categorize functionality by assigning secreted protein sequences to biological pathways. *Fc epsilon RI signaling pathway*, *T cell receptor signaling pathway*, *leukocyte transendothelial migration *and *chemokine signaling pathway *represent the immune system related pathways which could play a critical role in understanding the immune responses.

**Figure 3:**
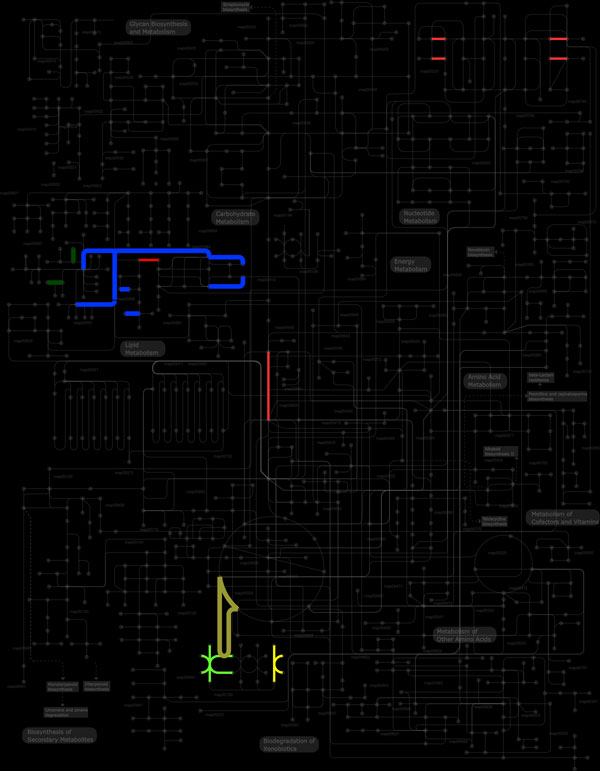
**Biological pathways mapped using iPath tool for putative excretory-secretory proteins**. The highlighted areas represent the pathways identified in the whole pathway.

We were also able to identify pathways related to neurodegenerative diseases and infectious diseases. Figure 3 shows the pathways represented using the ipath tool [125]. Identification of the role of such proteins as potential players in pathway analysis will help in our understanding of nematode biology in the context of parasite-host interplay. However, they are thought to be involved in immune responses in either the host or the parasite, which can be the focus of future studies. Of the pathways identified using KAAS, the protein family comprising serine, cysteine and metallo-proteinases and proteinase inhibitors in the EST datasets could form the basis of *in vitro *and *in vivo *studies. The parasite might be protected against digestive degradation by blocking endogenous proteinases within the host, with proteinase inhibitors. Tissue migration and other interactions with host cells may be facilitated by the function of these enzymes, by mediating or changing proteolytic functions [53]. Several studies have considered these enzymes as important therapeutic targets for parasite control [54-56,93]. Results from the pathway analysis carried out using KOBAS were compared with the results obtained using KAAS. The identification of domain/motif or region in a protein sequence characteristic for a particular protein family helps in the annotation by the assignment of protein function. We also searched the InterPro member databases [126] using Interproscan. Amongst the InterPro domains identified, the *Metridin-like ShK *and *transthyretin-like domains *were amongst the most represented, followed by *C-type lectin*, *saposin-like *and *SCP-like *extracellular domains. The *Metridin-like ShK *domain has already been shown to be highly represented in Strongylida and is often present in metallopeptidases [127,128]. The results showed that the most common molecules associated with the extracellular region correspond to *allergen V5/Tpx-1 related protein*. Additional File 14 contains the domain details of ES proteins. Overall, KOBAS and KAAS provided similar results.

Homologues RNAi phenotypes were identified by the comparison of 112 predicted ES proteins with the free-living nematode *C. elegans *and the associated RNAi phenotypes were studied to understand the function(s) and importance of homologous genes in other nematodes (of animals).

From these, 133 *C. elegans *homologues were retrieved with RNAi phenotypes (Additional File 15): *Emb *(embryonic lethal, including pleiotropic defects severe early emb), *Lva *(larval arrest), *Gro *(slow growth). *Stp *(sterile progeny), *Lvl *(larval lethal) and *Ste *(maternal sterile). In the current dataset, we have selected RNAi phenotypes essential for nematode survival or growth as well as those representing potential drug and/or vaccine targets [129,130]. Lethality can be considered as the most attractive RNAi phenotype applicable to all developmental stages that are less susceptible to available drugs as a result of interference with a vital process. Other attractive phenotypes include sterility that would lead to death. RNAi phenotypes help in understanding the concerns regarding genetic redundancy [131].

## Competing interests

The authors declare that they have no competing interests.

## Authors' contributions

MM generated and pre-processed the data. RM carried out the analysis, computational studies and drafted the manuscript. RM, SR and RBG participated in the design of the study and interpretation of data. SR, MM and RBG conceived the project and SR finalized the manuscript. All authors have read and approved the final manuscript.

## Supplementary Material

Additional File 1 **Comparison of rESTs from *Teladorsagia circumcincta *with *C. elegans***. Click here for file

Additional File 2 ***Teladorsagia circumcincta *homologues in Clade V of the phylum Nematoda, comprising *Haemonchus contortus*, *Necator americanus*, *Nippostrongylus brasiliensis*, *Ostertagia ostertagi*, *Pristionchus pacificus*, *Ancylostoma caninum*, *Ancylostoma ceylanicum *and *Dictyocaulus viviparus***. Click here for file

Additional File 3 **Metabolic pathways in *Teladorsagia circumcincta *mapped by Kyoto Encyclopedia of Genes and Genomes**. Click here for file

Additional File 4 **GO Annotation for proteins**. Click here for file

Additional File 5 **Secreted proteins predicted from rESTs from *Teladorsagia circumcincta *and their homologues and RNAi phenotypes**. Click here for file

Additional File 6 **Secretory proteins predicted from *Teladorsagia circumcincta *rESTs - comparison with proteomic data**. Click here for file

Additional File 7 **Transmembrane proteins predicted from rESTs from *Teladorsagia circumcincta *and their homologues and RNAi phenotypes**. Click here for file

Additional File 8 **GO Annotation for secreted proteins**. Click here for file

Additional File 9 **Pathway Analysis of secreted proteins using KOBAS**. Click here for file

Additional File 10 **Pathway Analysis of secreted proteins using KAAS**. Click here for file

Additional File 11 **Pathway analysis of secreted proteins obtained from SecretomeP**. Click here for file

Additional File 12 **Protein Domain Analysis in *Teladorsagia circumcincta***. Click here for file

Additional File 13 **Top NR description of *Teladorsagia circumcincta *protein sequences**. Click here for file

Additional File 14 **InterProScan analysis :representative protein domains/families**. Click here for file

Additional File 15 **Comparison of secreted proteins from *Teladorsagia circumcincta *with *C. elegans***. Click here for file

## References

[B1] Abubucker S Zarlenga DS Martin J Yin Y Wang Z McCarter JP Gasbarree L Wilson RK Mitreva M The transcriptomes of the cattle parasitic nematode Ostertagia ostartagi Veterinary parasitology 2009 162 1-2 89 99 10.1016/j.vetpar.2009.02.023 19346077 PMC2677129

[B2] O'Connor LJ Walkden-Brown SW Kahn LP Ecology of the free-living stages of major trichostrongylid parasites of sheep Vet Parasitol 2006 142 1-2 1 15 10.1016/j.vetpar.2006.08.035 17011129

[B3] Gibson TE Everett G Effect of different levels of intake of Ostertagia circumcincta larvae on the faecal egg counts and weight gain of lambs J Comp Pathol 1976 86 2 269 274 10.1016/0021-9975(76)90051-7 1270640

[B4] McNeilly TN Devaney E Matthews JB Teladorsagia circumcincta in the sheep abomasum: defining the role of dendritic cells in T cell regulation and protective immunity Parasite Immunol 2009 31 7 347 356 10.1111/j.1365-3024.2009.01110.x 19527450

[B5] Nisbet AJ Smith SK Armstrong S Meikle LI Wildblood LA Beynon RJ Matthews JB Teladorsagia circumcincta: activation-associated secreted proteins in excretory/secretory products of fourth stage larvae are targets of early IgA responses in infected sheep Exp Parasitol 2010 125 4 329 337 10.1016/j.exppara.2010.02.014 20206168

[B6] Dicker AJ Nath M Yaga R Nisbet AJ Lainson FA Gilleard JS Skuce PJ Teladorsagia circumcincta: the transcriptomic response of a multi-drug-resistant isolate to ivermectin exposure in vitro Exp Parasitol 2011 127 2 351 356 10.1016/j.exppara.2010.08.019 20816955

[B7] Hein WR Pernthaner A Piedrafita D Meeusen EN Immune mechanisms of resistance to gastrointestinal nematode infections in sheep Parasite Immunol 2010 32 8 541 548 20626809 10.1111/j.1365-3024.2010.01213.x

[B8] Hewitson JP Grainger JR Maizels RM Helminth immunoregulation: the role of parasite secreted proteins in modulating host immunity Mol Biochem Parasitol 2009 167 1 1 11 10.1016/j.molbiopara.2009.04.008 19406170 PMC2706953

[B9] Hotez PJ Bethony JM Diemert DJ Pearson M Loukas A Developing vaccines to combat hookworm infection and intestinal schistosomiasis Nat Rev Microbiol 2010 8 11 814 826 10.1038/nrmicro2438 20948553

[B10] De Vries E Bakker N Krijgsveld J Knox DP Heck AJ Yatsuda AP An AC-5 cathepsin B-like protease purified from Haemonchus contortus excretory secretory products shows protective antigen potential for lambs Vet Res 2009 40 4 41 10.1051/vetres/2009025 19401141 PMC2701184

[B11] Greub G Kebbi-Beghdadi C Bertelli C Collyn F Riederer BM Yersin C Croxatto A Raoult D High throughput sequencing and proteomics to identify immunogenic proteins of a new pathogen: the dirty genome approach PLoS One 2009 4 12 e8423 10.1371/journal.pone.0008423 20037647 PMC2793016

[B12] Smith SK Nisbet AJ Meikle LI Inglis NF Sales J Beynon RJ Matthews JB Proteomic analysis of excretory/secretory products released by Teladorsagia circumcincta larvae early post-infection Parasite Immunol 2009 31 1 10 19 10.1111/j.1365-3024.2008.01067.x 19121079

[B13] Nisbet AJ Zarlenga DS Knox DP Meikle LI Wildblood LA Matthews JB A calcium-activated apyrase from Teladorsagia circumcincta: an excretory/secretory antigen capable of modulating host immune responses? Parasite Immunol 2011 33 4 236 243 10.1111/j.1365-3024.2011.01278.x 21208222

[B14] Nisbet AJ Bell NE McNeilly TN Knox DP Maizels RM Meikle LI Wildblood LA Matthews JB A macrophage migration inhibitory factor-like tautomerase from Teladorsagia circumcincta (Nematoda: Strongylida) Parasite Immunol 2010 32 7 503 511 10.1111/j.1365-3024.2010.01215.x 20591121 PMC6485387

[B15] Ranganathan S Nagaraj SH Hu M Strube C Schnieder T Gasser RB A transcriptomic analysis of the adult stage of the bovine lungworm, Dictyocaulus viviparus BMC Genomics 2007 8 311 10.1186/1471-2164-8-311 17784965 PMC2131760

[B16] Nagaraj SH Gasser RB Nisbet AJ Ranganathan S In silico analysis of expressed sequence tags from Trichostrongylus vitrinus (Nematoda): comparison of the automated ESTExplorer workflow platform with conventional database searches BMC Bioinformatics 2008 9 Suppl 1 S10 10.1186/1471-2105-9-S1-S10 18315841 PMC2259411

[B17] Nisbet AJ Gasser RB Profiling of gender-specific gene expression for Trichostrongylus vitrinus (Nematoda: Strongylida) by microarray analysis of expressed sequence tag libraries constructed by suppressive-subtractive hybridisation Int J Parasitol 2004 34 5 633 643 10.1016/j.ijpara.2003.12.007 15064128

[B18] Cantacessi C Mitreva M Campbell BE Hall RS Young ND Jex AR Ranganathan S Gasser RB First transcriptomic analysis of the economically important parasitic nematode, Trichostrongylus colubriformis, using a next-generation sequencing approach Infect Genet Evol 2010 10 8 1199 1207 10.1016/j.meegid.2010.07.024 20692378 PMC3666958

[B19] Chen YA Lin CC Wang CD Wu HB Hwang PI An optimized procedure greatly improves EST vector contamination removal BMC Genomics 2007 8 416 10.1186/1471-2164-8-416 17997864 PMC2194723

[B20] Huang X Madan A CAP3: A DNA sequence assembly program Genome Res 1999 9 9 868 877 10.1101/gr.9.9.868 10508846 PMC310812

[B21] Iseli C Jongeneel CV Bucher P ESTScan: a program for detecting, evaluating, and reconstructing potential coding regions in EST sequences Proc Int Conf Intell Syst Mol Biol 1999 138 148 10786296

[B22] Quevillon E Silventoinen V Pillai S Harte N Mulder N Apweiler R Lopez R InterProScan: protein domains identifier Nucleic Acids Res 2005 33 Web Server W116 120 15980438 10.1093/nar/gki442 PMC1160203

[B23] Conesa A Gotz S Garcia-Gomez JM Terol J Talon M Robles M Blast2GO: a universal tool for annotation, visualization and analysis in functional genomics research Bioinformatics 2005 21 18 3674 3676 10.1093/bioinformatics/bti610 16081474

[B24] Wu J Mao X Cai T Luo J Wei L KOBAS server: a web-based platform for automated annotation and pathway identification Nucleic Acids Res 2006 34 Web Server W720 724 16845106 10.1093/nar/gkl167 PMC1538915

[B25] Kanehisa M Goto S Hattori M Aoki-Kinoshita KF Itoh M Kawashima S Katayama T Araki M Hirakawa M From genomics to chemical genomics: new developments in KEGG Nucleic Acids Res 2006 34 Database D354 357 16381885 10.1093/nar/gkj102 PMC1347464

[B26] Moriya Y Itoh M Okuda S Yoshizawa AC Kanehisa M KAAS: an automatic genome annotation and pathway reconstruction server Nucleic Acids Res 2007 35 Web Server W182 185 17526522 10.1093/nar/gkm321 PMC1933193

[B27] Parkinson J Blaxter M SimiTri--visualizing similarity relationships for groups of sequences Bioinformatics 2003 19 3 390 395 10.1093/bioinformatics/btf870 12584125

[B28] Bendtsen JD Nielsen H von Heijne G Brunak S Improved prediction of signal peptides: SignalP 3.0 J Mol Biol 2004 340 4 783 795 10.1016/j.jmb.2004.05.028 15223320

[B29] Bendtsen JD Jensen LJ Blom N Von Heijne G Brunak S Feature-based prediction of non-classical and leaderless protein secretion Protein Eng Des Sel 2004 17 4 349 356 10.1093/protein/gzh037 15115854

[B30] Emanuelsson O Brunak S von Heijne G Nielsen H Locating proteins in the cell using TargetP, SignalP and related tools Nat Protoc 2007 2 4 953 971 10.1038/nprot.2007.131 17446895

[B31] Bieri T Blasiar D Ozersky P Antoshechkin I Bastiani C Canaran P Chan J Chen N Chen WJ Davis P WormBase: new content and better access Nucleic Acids Res 2007 35 Database D506 510 17099234 10.1093/nar/gkl818 PMC1669750

[B32] Parkinson J Mitreva M Whitton C Thomson M Daub J Martin J Schmid R Hall N Barrell B Waterston RH A transcriptomic analysis of the phylum Nematoda Nat Genet 2004 36 12 1259 1267 10.1038/ng1472 15543149

[B33] Vercauteren I Geldhof P Peelaers I Claerebout E Berx G Vercruysse J Identification of excretory-secretory products of larval and adult Ostertagia ostertagi by immunoscreening of cDNA libraries Mol Biochem Parasitol 2003 126 2 201 208 10.1016/S0166-6851(02)00274-8 12615319

[B34] Nisbet AJ Redmond DL Matthews JB Watkins C Yaga R Jones JT Nath M Knox DP Stage-specific gene expression in Teladorsagia circumcincta (Nematoda: Strongylida) infective larvae and early parasitic stages Int J Parasitol 2008 38 7 829 838 10.1016/j.ijpara.2007.10.016 18062971

[B35] Henriksen A King TP Mirza O Monsalve RI Meno K Ipsen H Larsen JN Gajhede M Spangfort MD Major venom allergen of yellow jackets, Ves v 5: structural characterization of a pathogenesis-related protein superfamily Proteins 2001 45 4 438 448 10.1002/prot.1160 11746691

[B36] Lu G Villalba M Coscia MR Hoffman DR King TP Sequence analysis and antigenic cross-reactivity of a venom allergen, antigen 5, from hornets, wasps, and yellow jackets J Immunol 1993 150 7 2823 2830 8454859

[B37] Cottee PA Nisbet AJ Abs El-Osta YG Webster TL Gasser RB Construction of gender-enriched cDNA archives for adult Oesophagostomum dentatum by suppressive-subtractive hybridization and a microarray analysis of expressed sequence tags Parasitology 2006 132 Pt 5 691 708 16426483 10.1017/S0031182005009728

[B38] Benson DA Karsch-Mizrachi I Lipman DJ Ostell J Sayers EW GenBank Nucleic Acids Res 2012 39 Database D32 37 10.1093/nar/gkq1079 PMC3013681 21071399

[B39] http://wormbase.org

[B40] Huang R Wallqvist A Covell DG Comprehensive analysis of pathway or functionally related gene expression in the National Cancer Institute's anticancer screen Genomics 2006 87 3 315 328 10.1016/j.ygeno.2005.11.011 16386875

[B41] Craig H Isaac RE Brooks DR Unravelling the moulting degradome: new opportunities for chemotherapy? Trends Parasitol 2007 23 6 248 253 10.1016/j.pt.2007.04.003 17459772

[B42] Bennuru S Semnani R Meng Z Ribeiro JM Veenstra TD Nutman TB Brugia malayi excreted/secreted proteins at the host/parasite interface: stage- and gender-specific proteomic profiling PLoS Negl Trop Dis 2009 3 4 e410 10.1371/journal.pntd.0000410 19352421 PMC2659452

[B43] Hong X Bouvier J Wong MM Yamagata GY McKerrow JH Brugia pahangi: identification and characterization of an aminopeptidase associated with larval molting Exp Parasitol 1993 76 2 127 133 10.1006/expr.1993.1015 8454021

[B44] Rhoads ML Fetterer RH Urban JF Jr Secretion of an aminopeptidase during transition of third- to fourth-stage larvae of Ascaris suum J Parasitol 1997 83 5 780 784 10.2307/3284267 9379278

[B45] Rhoads ML Fetterer RH Urban JF Jr Effect of protease class-specific inhibitors on in vitro development of the third- to fourth-stage larvae of Ascaris suum J Parasitol 1998 84 4 686 690 10.2307/3284570 9714194

[B46] Patterson GI Padgett RW TGF beta-related pathways. Roles in Caenorhabditis elegans development Trends Genet 2000 16 1 27 33 10.1016/S0168-9525(99)01916-2 10637628

[B47] Beall MJ Pearce EJ Transforming growth factor-beta and insulin-like signalling pathways in parasitic helminths Int J Parasitol 2002 32 4 399 404 10.1016/S0020-7519(01)00348-4 11849636

[B48] Ren P Lim CS Johnsen R Albert PS Pilgrim D Riddle DL Control of C. elegans larval development by neuronal expression of a TGF-beta homolog Science 1996 274 5291 1389 1391 10.1126/science.274.5291.1389 8910282

[B49] Sze JY Victor M Loer C Shi Y Ruvkun G Food and metabolic signalling defects in a Caenorhabditis elegans serotonin-synthesis mutant Nature 2000 403 6769 560 564 10.1038/35000609 10676966

[B50] Gomez-Escobar N van den Biggelaar A Maizels R A member of the TGF-beta receptor gene family in the parasitic nematode Brugia pahangi Gene 1997 199 1-2 101 109 10.1016/S0378-1119(97)00353-3 9358045

[B51] Gomez-Escobar N Gregory WF Maizels RM Identification of tgh-2, a filarial nematode homolog of Caenorhabditis elegans daf-7 and human transforming growth factor beta, expressed in microfilarial and adult stages of Brugia malayi Infect Immun 2000 68 11 6402 6410 10.1128/IAI.68.11.6402-6410.2000 11035752 PMC97726

[B52] Crook M Thompson FJ Grant WN Viney ME daf-7 and the development of Strongyloides ratti and Parastrongyloides trichosuri Mol Biochem Parasitol 2005 139 2 213 223 10.1016/j.molbiopara.2004.11.010 15664656

[B53] Knox DP Proteinase inhibitors and helminth parasite infection Parasite Immunol 2007 29 2 57 71 17241394 10.1111/j.1365-3024.2006.00913.x

[B54] Karanu FN Rurangirwa FR McGuire TC Jasmer DP Haemonchus contortus: identification of proteases with diverse characteristics in adult worm excretory-secretory products Exp Parasitol 1993 77 3 362 371 10.1006/expr.1993.1093 8224091

[B55] Kovaleva ES Masler EP Skantar AM Chitwood DJ Novel matrix metalloproteinase from the cyst nematodes Heterodera glycines and Globodera rostochiensis Mol Biochem Parasitol 2004 136 1 109 112 10.1016/j.molbiopara.2004.03.001 15138072

[B56] Yatsuda AP Bakker N Krijgsveld J Knox DP Heck AJ de Vries E Identification of secreted cysteine proteases from the parasitic nematode Haemonchus contortus detected by biotinylated inhibitors Infect Immun 2006 74 3 1989 1993 10.1128/IAI.74.3.1989-1993.2006 16495580 PMC1418636

[B57] Clarke ND Berg JM Zinc fingers in Caenorhabditis elegans: finding families and probing pathways Science 1998 282 5396 2018 2022 9851917 10.1126/science.282.5396.2018

[B58] Plowman GD Sudarsanam S Bingham J Whyte D Hunter T The protein kinases of Caenorhabditis elegans: a model for signal transduction in multicellular organisms Proc Natl Acad Sci USA 1999 96 24 13603 13610 10.1073/pnas.96.24.13603 10570119 PMC24111

[B59] Chalmers IW McArdle AJ Coulson RM Wagner MA Schmid R Hirai H Hoffmann KF Developmentally regulated expression, alternative splicing and distinct sub-groupings in members of the Schistosoma mansoni venom allergen-like (SmVAL) gene family BMC Genomics 2008 9 89 10.1186/1471-2164-9-89 18294395 PMC2270263

[B60] Vermeire JJ Cho Y Lolis E Bucala R Cappello M Orthologs of macrophage migration inhibitory factor from parasitic nematodes Trends Parasitol 2008 24 8 355 363 10.1016/j.pt.2008.04.007 18603473 PMC3615561

[B61] Cantacessi C Campbell BE Visser A Geldhof P Nolan MJ Nisbet AJ Matthews JB Loukas A Hofmann A Otranto D A portrait of the "SCP/TAPS" proteins of eukaryotes--developing a framework for fundamental research and biotechnological outcomes Biotechnol Adv 2009 27 4 376 388 10.1016/j.biotechadv.2009.02.005 19239923

[B62] Cantacessi C Campbell BE Young ND Jex AR Hall RS Presidente PJ Zawadzki JL Zhong W Aleman-Meza B Loukas A Differences in transcription between free-living and CO2-activated third-stage larvae of Haemonchus contortus BMC Genomics 2010 11 266 10.1186/1471-2164-11-266 20420710 PMC2880303

[B63] McCarter JP Mitreva MD Martin J Dante M Wylie T Rao U Pape D Bowers Y Theising B Murphy CV Analysis and functional classification of transcripts from the nematode Meloidogyne incognita Genome Biol 2003 4 4 R26 10.1186/gb-2003-4-4-r26 12702207 PMC154577

[B64] Hewitson JP Harcus YM Curwen RS Dowle AA Atmadja AK Ashton PD Wilson A Maizels RM The secretome of the filarial parasite, Brugia malayi: proteomic profile of adult excretory-secretory products Mol Biochem Parasitol 2008 160 1 8 21 10.1016/j.molbiopara.2008.02.007 18439691

[B65] McMahon SA Miller JL Lawton JA Kerkow DE Hodes A Marti-Renom MA Doulatov S Narayanan E Sali A Miller JF The C-type lectin fold as an evolutionary solution for massive sequence variation Nat Struct Mol Biol 2005 12 10 886 892 10.1038/nsmb992 16170324

[B66] Loukas A Maizels RM Helminth C-type lectins and host-parasite interactions Parasitol Today 2000 16 8 333 339 10.1016/S0169-4758(00)01704-X 10900481

[B67] McElwee JJ Schuster E Blanc E Thomas JH Gems D Shared transcriptional signature in Caenorhabditis elegans Dauer larvae and long-lived daf-2 mutants implicates detoxification system in longevity assurance J Biol Chem 2004 279 43 44533 44543 10.1074/jbc.M406207200 15308663

[B68] Wolf DA Jackson PK Cell cycle: oiling the gears of anaphase Curr Biol 1998 8 18 R636 639 10.1016/S0960-9822(07)00410-1 9740795

[B69] Leipe DD Koonin EV Aravind L STAND, a class of P-loop NTPases including animal and plant regulators of programmed cell death: multiple, complex domain architectures, unusual phyletic patterns, and evolution by horizontal gene transfer J Mol Biol 2004 343 1 1 28 10.1016/j.jmb.2004.08.023 15381417

[B70] Hartman D Cottee PA Savin KW Bhave M Presidente PJ Fulton L Walkiewicz M Newton SE Haemonchus contortus: molecular characterisation of a small heat shock protein Exp Parasitol 2003 104 3-4 96 103 10.1016/S0014-4894(03)00138-3 14552856

[B71] Nagamune K Moreno SN Chini EN Sibley LD Calcium regulation and signaling in apicomplexan parasites Subcell Biochem 2008 47 70 81 10.1007/978-0-387-78267-6_5 18512342

[B72] Fujiwara RT Cancado GG Freitas PA Santiago HC Massara CL Dos Santos Carvalho O Correa-Oliveira R Geiger SM Bethony J Necator americanus infection: a possible cause of altered dendritic cell differentiation and eosinophil profile in chronically infected individuals PLoS Negl Trop Dis 2009 3 3 e399 10.1371/journal.pntd.0000399 19308259 PMC2654967

[B73] Estevez AO Cowie RH Gardner KL Estevez M Both insulin and calcium channel signaling are required for developmental regulation of serotonin synthesis in the chemosensory ADF neurons of Caenorhabditis elegans Dev Biol 2006 298 1 32 44 10.1016/j.ydbio.2006.06.005 16860310

[B74] Roberts TM Stewart M Acting like actin. The dynamics of the nematode major sperm protein (msp) cytoskeleton indicate a push-pull mechanism for amoeboid cell motility J Cell Biol 2000 149 1 7 12 10.1083/jcb.149.1.7 10747081 PMC2175093

[B75] Buttery SM Ekman GC Seavy M Stewart M Roberts TM Dissection of the Ascaris sperm motility machinery identifies key proteins involved in major sperm protein-based amoeboid locomotion Mol Biol Cell 2003 14 12 5082 5088 10.1091/mbc.E03-04-0246 14565983 PMC284809

[B76] Strube C Buschbaum S Schnieder T Molecular characterization and real-time PCR transcriptional analysis of Dictyocaulus viviparus major sperm proteins Parasitol Res 2009 104 3 543 551 10.1007/s00436-008-1228-5 18853187

[B77] Williamson AL Brindley PJ Knox DP Hotez PJ Loukas A Digestive proteases of blood-feeding nematodes Trends Parasitol 2003 19 9 417 423 10.1016/S1471-4922(03)00189-2 12957519

[B78] Williamson AL Lecchi P Turk BE Choe Y Hotez PJ McKerrow JH Cantley LC Sajid M Craik CS Loukas A A multi-enzyme cascade of hemoglobin proteolysis in the intestine of blood-feeding hookworms J Biol Chem 2004 279 34 35950 35957 10.1074/jbc.M405842200 15199048

[B79] Ranjit N Zhan B Stenzel DJ Mulvenna J Fujiwara R Hotez PJ Loukas A A family of cathepsin B cysteine proteases expressed in the gut of the human hookworm, Necator americanus Mol Biochem Parasitol 2008 160 2 90 99 10.1016/j.molbiopara.2008.04.008 18501979

[B80] Hotez P Haggerty J Hawdon J Milstone L Gamble HR Schad G Richards F Metalloproteases of infective Ancylostoma hookworm larvae and their possible functions in tissue invasion and ecdysis Infect Immun 1990 58 12 3883 3892 2254016 10.1128/iai.58.12.3883-3892.1990 PMC313750

[B81] Williamson AL Lustigman S Oksov Y Deumic V Plieskatt J Mendez S Zhan B Bottazzi ME Hotez PJ Loukas A Ancylostoma caninum MTP-1, an astacin-like metalloprotease secreted by infective hookworm larvae, is involved in tissue migration Infect Immun 2006 74 2 961 967 10.1128/IAI.74.2.961-967.2006 16428741 PMC1360348

[B82] Hotez PJ Ashcom J Zhan B Bethony J Loukas A Hawdon J Wang Y Jin Q Jones KC Dobardzic A Effect of vaccination with a recombinant fusion protein encoding an astacinlike metalloprotease (MTP-1) secreted by host-stimulated Ancylostoma caninum third-stage infective larvae J Parasitol 2003 89 4 853 855 10.1645/GE-46R 14533704

[B83] Borchert N Becker-Pauly C Wagner A Fischer P Stocker W Brattig NW Identification and characterization of onchoastacin, an astacin-like metalloproteinase from the filaria Onchocerca volvulus Microbes Infect 2007 9 4 498 506 10.1016/j.micinf.2007.01.007 17347015

[B84] Skach WR The expanding role of the ER translocon in membrane protein folding J Cell Biol 2007 179 7 1333 1335 10.1083/jcb.200711107 18166647 PMC2373491

[B85] Gems D Ferguson CJ Robertson BD Nieves R Page AP Blaxter ML Maizels RM An abundant, trans-spliced mRNA from Toxocara canis infective larvae encodes a 26-kDa protein with homology to phosphatidylethanolamine-binding proteins J Biol Chem 1995 270 31 18517 18522 10.1074/jbc.270.31.18517 7629180

[B86] Maizels RM Tetteh KK Loukas A Toxocara canis: genes expressed by the arrested infective larval stage of a parasitic nematode Int J Parasitol 2000 30 4 495 508 10.1016/S0020-7519(00)00022-9 10731572

[B87] Loukas A Hintz M Linder D Mullin NP Parkinson J Tetteh KK Maizels RM A family of secreted mucins from the parasitic nematode Toxocara canis bears diverse mucin domains but shares similar flanking six-cysteine repeat motifs J Biol Chem 2000 275 50 39600 39607 10.1074/jbc.M005632200 10950959

[B88] Daub J Loukas A Pritchard DI Blaxter M A survey of genes expressed in adults of the human hookworm, Necator americanus Parasitology 2000 120 Pt 2 171 184 10726278 10.1017/s0031182099005375

[B89] De Maere V Vercauteren I Saverwyns H Claerebout E Berx G Vercruysse J Identification of potential protective antigens of Ostertagia ostertagi with local antibody probes Parasitology 2002 125 Pt 4 383 391 12403327 10.1017/s0031182002002196

[B90] Blaxter M Caenorhabditis elegans is a nematode Science 1998 282 5396 2041 2046 9851921 10.1126/science.282.5396.2041

[B91] Sonnhammer EL Durbin R Analysis of protein domain families in Caenorhabditis elegans Genomics 1997 46 2 200 216 10.1006/geno.1997.4989 9417907

[B92] Saverwyns H Visser A Van Durme J Power D Morgado I Kennedy MW Knox DP Schymkowitz J Rousseau F Gevaert K Analysis of the transthyretin-like (TTL) gene family in Ostertagia ostertagi--comparison with other strongylid nematodes and Caenorhabditis elegans Int J Parasitol 2008 38 13 1545 1556 10.1016/j.ijpara.2008.04.004 18571174

[B93] Nagaraj SH Gasser RB Ranganathan S Needles in the EST haystack: large-scale identification and analysis of excretory-secretory (ES) proteins in parasitic nematodes using expressed sequence tags (ESTs) PLoS Negl Trop Dis 2008 2 9 e301 10.1371/journal.pntd.0000301 18820748 PMC2553489

[B94] Jacob J Vanholme B Haegeman A Gheysen G Four transthyretin-like genes of the migratory plant-parasitic nematode Radopholus similis: members of an extensive nematode-specific family Gene 2007 402 1-2 9 19 10.1016/j.gene.2007.07.015 17765408

[B95] Bruhn H A short guided tour through functional and structural features of saposin-like proteins Biochem J 2005 389 Pt 2 249 257 15992358 10.1042/BJ20050051 PMC1175101

[B96] Leippe M Andra J Nickel R Tannich E Muller-Eberhard HJ Amoebapores, a family of membranolytic peptides from cytoplasmic granules of Entamoeba histolytica: isolation, primary structure, and pore formation in bacterial cytoplasmic membranes Mol Microbiol 1994 14 5 895 904 10.1111/j.1365-2958.1994.tb01325.x 7715451

[B97] Andersson M Gunne H Agerberth B Boman A Bergman T Sillard R Jornvall H Mutt V Olsson B Wigzell H NK-lysin, a novel effector peptide of cytotoxic T and NK cells. Structure and cDNA cloning of the porcine form, induction by interleukin 2, antibacterial and antitumour activity Embo J 1995 14 8 1615 1625 7737114 10.1002/j.1460-2075.1995.tb07150.x PMC398254

[B98] Zhai Y Saier MH Jr The amoebapore superfamily Biochim Biophys Acta 2000 1469 2 87 99 10.1016/S0304-4157(00)00003-4 10998571

[B99] Don TA Oksov Y Lustigman S Loukas A Saposin-like proteins from the intestine of the blood-feeding hookworm, Ancylostoma caninum Parasitology 2007 134 Pt 3 427 436 17109779 10.1017/S003118200600148X

[B100] Harcus Y Nicoll G Murray J Filbey K Gomez-Escobar N Maizels RM C-type lectins from the nematode parasites Heligmosomoides polygyrus and Nippostrongylus brasiliensis Parasitol Int 2009 58 4 461 470 10.1016/j.parint.2009.08.011 19751847 PMC2792708

[B101] Craig H Wastling JM Knox DP A preliminary proteomic survey of the in vitro excretory/secretory products of fourth-stage larval and adult Teladorsagia circumcincta Parasitology 2006 132 Pt 4 535 543 16388693 10.1017/S0031182005009510

[B102] Hotez PJ Zhan B Bethony JM Loukas A Williamson A Goud GN Hawdon JM Dobardzic A Dobardzic R Ghosh K Progress in the development of a recombinant vaccine for human hookworm disease: the Human Hookworm Vaccine Initiative Int J Parasitol 2003 33 11 1245 1258 10.1016/S0020-7519(03)00158-9 13678639

[B103] Visser A Van Zeveren AM Meyvis Y Peelaers I Van den Broeck W Gevaert K Vercruysse J Claerebout E Geldhof P Gender-enriched transcription of activation associated secreted proteins in Ostertagia ostertagi Int J Parasitol 2008 38 3-4 455 465 10.1016/j.ijpara.2007.08.008 17961575

[B104] Bin Z Hawdon J Qiang S Hainan R Huiqing Q Wei H Shu-Hua X Tiehua L Xing G Zheng F Ancylostoma secreted protein 1 (ASP-1) homologues in human hookworms Mol Biochem Parasitol 1999 98 1 143 149 10.1016/S0166-6851(98)00157-1 10029316

[B105] Moreno Y Geary TG Stage- and gender-specific proteomic analysis of Brugia malayi excretory-secretory products PLoS Negl Trop Dis 2008 2 10 e326 10.1371/journal.pntd.0000326 18958170 PMC2569413

[B106] Murray J Gregory WF Gomez-Escobar N Atmadja AK Maizels RM Expression and immune recognition of Brugia malayi VAL-1, a homologue of vespid venom allergens and Ancylostoma secreted proteins Mol Biochem Parasitol 2001 118 1 89 96 10.1016/S0166-6851(01)00374-7 11704277

[B107] Schallig HD van Leeuwen MA Verstrepen BE Cornelissen AW Molecular characterization and expression of two putative protective excretory secretory proteins of Haemonchus contortus Mol Biochem Parasitol 1997 88 1-2 203 213 10.1016/S0166-6851(97)00093-5 9274880

[B108] Yatsuda AP Krijgsveld J Cornelissen AW Heck AJ de Vries E Comprehensive analysis of the secreted proteins of the parasite Haemonchus contortus reveals extensive sequence variation and differential immune recognition J Biol Chem 2003 278 19 16941 16951 10.1074/jbc.M212453200 12576473

[B109] Cass CL Johnson JR Califf LL Xu T Hernandez HJ Stadecker MJ Yates JR Williams DL Proteomic analysis of Schistosoma mansoni egg secretions Mol Biochem Parasitol 2007 155 2 84 93 10.1016/j.molbiopara.2007.06.002 17644200 PMC2077830

[B110] Curwen RS Ashton PD Sundaralingam S Wilson RA Identification of novel proteases and immunomodulators in the secretions of schistosome cercariae that facilitate host entry Mol Cell Proteomics 2006 5 5 835 844 10.1074/mcp.M500313-MCP200 16469760

[B111] Maizels RM Gomez-Escobar N Gregory WF Murray J Zang X Immune evasion genes from filarial nematodes Int J Parasitol 2001 31 9 889 898 10.1016/S0020-7519(01)00213-2 11406138

[B112] Hawdon JM Jones BF Hoffman DR Hotez PJ Cloning and characterization of Ancylostoma-secreted protein. A novel protein associated with the transition to parasitism by infective hookworm larvae J Biol Chem 1996 271 12 6672 6678 10.1074/jbc.271.12.6672 8636085

[B113] Bower MA Constant SL Mendez S Necator americanus: the Na-ASP-2 protein secreted by the infective larvae induces neutrophil recruitment in vivo and in vitro Exp Parasitol 2008 118 4 569 575 10.1016/j.exppara.2007.11.014 18199436 PMC2359483

[B114] Ghosh K Hotez PJ Antibody-dependent reductions in mouse hookworm burden after vaccination with Ancylostoma caninum secreted protein 1 J Infect Dis 1999 180 5 1674 1681 10.1086/315059 10515831

[B115] Asojo OA Goud G Dhar K Loukas A Zhan B Deumic V Liu S Borgstahl GE Hotez PJ X-ray structure of Na-ASP-2, a pathogenesis-related-1 protein from the nematode parasite, Necator americanus, and a vaccine antigen for human hookworm infection J Mol Biol 2005 346 3 801 814 10.1016/j.jmb.2004.12.023 15713464

[B116] Zhan B Liu Y Badamchian M Williamson A Feng J Loukas A Hawdon JM Hotez PJ Molecular characterisation of the Ancylostoma-secreted protein family from the adult stage of Ancylostoma caninum Int J Parasitol 2003 33 9 897 907 10.1016/S0020-7519(03)00111-5 12906874

[B117] Tawe W Pearlman E Unnasch TR Lustigman S Angiogenic activity of Onchocerca volvulus recombinant proteins similar to vespid venom antigen 5 Mol Biochem Parasitol 2000 109 2 91 99 10.1016/S0166-6851(00)00231-0 10960168

[B118] Wang J Kim SK Global analysis of dauer gene expression in Caenorhabditis elegans Development 2003 130 8 1621 1634 10.1242/dev.00363 12620986

[B119] Moore J Tetley L Devaney E Identification of abundant mRNAs from the third stage larvae of the parasitic nematode, ostertagia ostertagi Biochem J 2000 347 Pt 3 763 770 10769181 PMC1221014

[B120] Zhan B Hotez PJ Wang Y Hawdon JM A developmentally regulated metalloprotease secreted by host-stimulated Ancylostoma caninum third-stage infective larvae is a member of the astacin family of proteases Mol Biochem Parasitol 2002 120 2 291 296 10.1016/S0166-6851(01)00453-4 11897134

[B121] Culley FJ Brown A Conroy DM Sabroe I Pritchard DI Williams TJ Eotaxin is specifically cleaved by hookworm metalloproteases preventing its action in vitro and in vivo J Immunol 2000 165 11 6447 6453 11086084 10.4049/jimmunol.165.11.6447

[B122] Blelloch R Kimble J Control of organ shape by a secreted metalloprotease in the nematode Caenorhabditis elegans Nature 1999 399 6736 586 590 10.1038/21196 10376599

[B123] Geldhof P Claerebout E Knox DP Jagneessens J Vercruysse J Proteinases released in vitro by the parasitic stages of the bovine abomasal nematode Ostertagia ostertagi Parasitology 2000 121 Pt 6 639 647 11155935 10.1017/s0031182000006806

[B124] Hawdon JM Jones BF Perregaux MA Hotez PJ Ancylostoma caninum: metalloprotease release coincides with activation of infective larvae in vitro Exp Parasitol 1995 80 2 205 211 10.1006/expr.1995.1025 7895832

[B125] Letunic I Yamada T Kanehisa M Bork P iPath: interactive exploration of biochemical pathways and networks Trends Biochem Sci 2008 33 3 101 103 10.1016/j.tibs.2008.01.001 18276143

[B126] Mulder NJ Apweiler R Attwood TK Bairoch A Bateman A Binns D Bork P Buillard V Cerutti L Copley R New developments in the InterPro database Nucleic Acids Res 2007 35 Database D224 228 17202162 10.1093/nar/gkl841 PMC1899100

[B127] Rawlings ND Barrett AJ Evolutionary families of metallopeptidases Methods Enzymol 1995 248 183 228 7674922 10.1016/0076-6879(95)48015-3

[B128] Mohrlen F Hutter H Zwilling R The astacin protein family in Caenorhabditis elegans Eur J Biochem 2003 270 24 4909 4920 10.1046/j.1432-1033.2003.03891.x 14653817

[B129] Geldhof P Visser A Clark D Saunders G Britton C Gilleard J Berriman M Knox D RNA interference in parasitic helminths: current situation, potential pitfalls and future prospects Parasitology 2007 134 Pt 5 609 619 17201997 10.1017/S0031182006002071

[B130] Kumar S Chaudhary K Foster JM Novelli JF Zhang Y Wang S Spiro D Ghedin E Carlow CK Mining predicted essential genes of Brugia malayi for nematode drug targets PLoS One 2007 2 11 e1189 10.1371/journal.pone.0001189 18000556 PMC2063515

[B131] Foster JM Zhang Y Kumar S Carlow CK Mining nematode genome data for novel drug targets Trends Parasitol 2005 21 3 101 104 10.1016/j.pt.2004.12.002 15734654

